# Wiggle—Predicting Functionally Flexible Regions from Primary Sequence

**DOI:** 10.1371/journal.pcbi.0020090

**Published:** 2006-07-14

**Authors:** Jenny Gu, Michael Gribskov, Philip E Bourne

**Affiliations:** 1 Department of Pharmacology and Biomedical Sciences Graduate Program, University of California San Diego, La Jolla, California, United States of America; 2 San Diego Supercomputer Center, University of California San Diego, La Jolla, California, United States of America; 3 Department of Biological Sciences, Purdue University, West Lafayette, Indiana, United States of America; Harvard University, United States of America

## Abstract

The Wiggle series are support vector machine–based predictors that identify regions of functional flexibility using only protein sequence information. Functionally flexible regions are defined as regions that can adopt different conformational states and are assumed to be necessary for bioactivity. Many advances have been made in understanding the relationship between protein sequence and structure. This work contributes to those efforts by making strides to understand the relationship between protein sequence and flexibility. A coarse-grained protein dynamic modeling approach was used to generate the dataset required for support vector machine training. We define our regions of interest based on the participation of residues in correlated large-scale fluctuations. Even with this structure-based approach to computationally define regions of functional flexibility, predictors successfully extract sequence-flexibility relationships that have been experimentally confirmed to be functionally important. Thus, a sequence-based tool to identify flexible regions important for protein function has been created. The ability to identify functional flexibility using a sequence based approach complements structure-based definitions and will be especially useful for the large majority of proteins with unknown structures. The methodology offers promise to identify structural genomics targets amenable to crystallization and the possibility to engineer more flexible or rigid regions within proteins to modify their bioactivity.

## Synopsis

Proteins are not static entities in biology and are constantly changing their shape and form to perform their necessary biological roles. While we are intuitively aware of their constantly changing nature, we have little understanding of how their flexibility is encoded in the protein sequence. To address this knowledge gap, predictors were created to identify sequence patterns that dictate local regions to be flexible and serve a functional purpose. By combining protein dynamic modeling and machine learning techniques, the Wiggle predictor series were able to generalize the sequence-flexibility relationship for all proteins. With these predictors we are able to identify flexible regions of functional importance such as hinges, recognition loops, and catalytic loops using only sequence information. This work has important contributions to our understanding of the sequence-flexibility relationship and paves the road to identifying local sequence modulations that impact protein function without necessarily changing the structure.

## Introduction

Protein structures are not rigid bodies, as suggested by time-independent solid-state crystal structures. Rather, proteins are selected by nature to balance between stability and flexibility in order to traverse the funnels of the protein energy landscape that characterize the conformational states needed to achieve a specific bioactivity. In part because of the way protein structures are traditionally represented and visualized in the crystallographic structure, the dynamics of protein motion is poorly conveyed and often neglected as the protein is treated as a static entity, although intuitively we know otherwise. Furthermore, protein sequence-structure relationships have been heavily focused on creating the most stable structure that may not necessarily be optimal for the execution or regulation of protein function. If the sequence is deterministic of the adopted protein fold, then the flexibility and dynamics of proteins should also be encoded by the sequence. Support for this notion comes from the previous demonstration that large amplitude fluctuations are mostly related to the overall protein shape [1,2], which in turn is defined by the sequence. In this work we develop a computational methodology that takes a small, but significant, step in understanding sequence-flexibility relationships important for protein function.

The flexibility of proteins is a necessary property to allow for conformational changes observed in allosteric interactions. The classic definition of allostery is the regulation of enzymes through the binding of effector molecules. This definition is now expanded to define allostery as the consequence of the redistribution of conformational states in the protein in response to a given external stimulus [3]. We are particularly interested in the contribution of entropy as an allosteric mechanism used by proteins to allow for these conformational shifts to occur [4,5] and how this feature may be encoded at the protein sequence level.

The Cooper-Dryden model of allostery is a theory that addresses the contribution of entropy to the allosteric free energy. In extreme cases, this theory suggests that allostery can be achieved in the absence of structural change by simply shifting the internal vibrational modes when reacting to an external stimulus such as ligand binding [6]. Associated with this model is the idea of remote entropy compensation, a scenario where a local entropy decrease in one area of a protein is compensated by an increase in entropy in another area. These regions can be located distantly from each other, thereby making the entropy compensation a long-range effect.

Entropy compensation has been observed using both computational and experimental approaches in many different proteins. Here we consider five examples to make the point. First, molecular dynamic (MD) simulations of lysozyme show differences between the dynamics of substrate bound and free states. When lysozyme is in complex with the substrate, a distant loop (residues 67 to 88) increases in fluctuation to compensate for the decreasing fluctuation observed for the substrate-contacting loop (residues 101 to 107) [7]. Second, global structural changes resulting from changes in local fluctuation induced by proton binding are observed in staphylococcal nucleases [8]. Third, spectroscopic experiments on the Tet repressor examined the fluorescence anisotropy decay of tryptophans introduced into a functionally important loop located distantly from the site of substrate binding. An increase in fluctuation was observed in this loop when anhydrotetracycline was bound to the Tet repressor [9]. Fourth, entropy compensation can be inferred from comparing X-ray structures of adenylate kinase in different conformations [10]. Fluctuations localized at the nucleoside monophosphate binding and LID domains, the substrate binding interface, show an inverse relationship with the fluctuations of loops α4-β3 and α5-β4 that are located distantly. Finally, mutational studies show long-range dynamic perturbations in eglin C detected by NMR. This protein is considered to be classically nonallosteric, an example where distant fluctuations can be affected by changes in sequence [11], a point we come back to subsequently.

In each of these cases, local regions of protein structure serve to accommodate the redistribution of vibrational modes and provide an energy reserve of allosteric free energy as proposed by the Cooper-Dryden model. The relaxation and tensing of regions of local structure is a transition from an ordered to disordered state and vice versa. Such local regions include hinges, recognition loops, and certain catalytic loops whose vibrational states change in the presence of an external stimulus such as substrate binding. While structurally dissimilar, hinges, recognition loops, and catalytic loops all exhibit characteristic fluctuations that differ from the mean fluctuation. Hinges are relatively immobile at the hinge point compared to surrounding fluctuations about the hinge, whereas recognition loops and, in certain examples, catalytic loops show minimal fluctuations at the extremities and maximal fluctuations at the center of the loop. We attempt to identify these regions based on the scale and cooperativeness of fluctuations that often define protein function and refer to them as *functionally flexible regions* (FFRs).

In this paper, we begin with a structure-based definition of an FFR to obtain our training dataset and describe prediction tools created as a result to identify these regions using only protein sequence information. With the growth of protein structures fueled by structural genomics [12], it is possible to generate a training dataset to begin efforts to understand relationships between protein sequence and functional flexibility (FF). First, we devised a method using protein dynamics modeling to identify FFRs using only a single protein conformation. Then we use machine learning techniques to identify protein regions with the measured amount of flexibility needed for bioactivity without using structural information. Overlaps are expected with existing disorder and order predictions since FFRs can exist in both states. Disordered predictors are trained to predict regions of high flexibility based on temperature factor information or regions of the protein with no electron density. Sequence analyses of predicted disordered regions have revealed the existence of different types of disorder [13] which may include FFRs. FFRs can also adopt ordered structures when triggered to do so under the right conditions.

The long-term goal of work such as this is to provide a generalized relationship between sequence and FF for all proteins. An immediate benefit would be in facilitating the structure solution process such that proteins less tractable for crystallization could be identified. Further, by our definition, FFRs border on forming an ordered structure; therefore, if such regions can be identified, it may be possible to introduce a few mutations to stabilize local regions that are not located on the ends of the polypeptide chain. This strategy has been utilized to successfully create a soluble analog of erythropoietin [14]. We also hope to contribute to the field of de novo protein design with the understanding of the relationship between protein sequence and dynamics. Recently, a three-dimensional structure unseen in nature with a root-mean-square deviation of 1.2 Å from the design model was engineered [15]. Conceivably, understanding sequence-flexibility relationships would be useful in guiding the engineering necessary to introduce the flexibility required for bioactivity in these newly designed proteins. Furthermore, as in the example of eglin C, there are flexibility modulating regions that cannot be obviously identified with structural inspection but possibly detected with improved understanding of sequence-flexibility relationships.

## Results/Discussion

### Case Studies of FFRs: Identification with an FF Score

We define FFRs to have the property of coordinated participation in large amplitude fluctuations that are different from the mean vibrational fluctuation of the protein. The Gaussian network model (GNM) [16], a coarse-grained protein dynamics modeling approach, was chosen to obtain fluctuation mode information needed to identify regions of interest because it is a computationally practical alternative to an all-atom MD simulation yet provides a good approximation of near-native protein fluctuation at longer time scales [17]. Classic all-atom MD provides accurate, detailed descriptions of molecular motion. However, simulations are limited by computational demands to a few tens of nanoseconds. GNM is able to address large-scale fluctuations that extend beyond the time scale of MD simulation, a capability important for some types of molecular recognition and allosteric rearrangements occurring at time scales of microseconds and longer. While GNM provides only an approximation, several studies comparing coarse-grained approaches to MD have shown that it is an accurate and efficient alternative [16–20].

There are two reasons for using protein dynamic modeling results instead of experimental temperature factors to define our target regions. First, by using protein dynamic modeling simulation, we are able to investigate protein flexibility with the added dimensionality of having functional importance. Second, by using modes of motion to define our target regions, we are able to focus specifically on large-amplitude fluctuations without including contributions from higher frequency fluctuations. These two features are the distinguishing qualities that set our predictors apart from other disorder predictors. The advantages of using this approach will be highlighted in the subsequent discussion and reflected in comparisons made to other disorder predictors.

To identify FFRs, we focus on the first two vibrational modes of protein fluctuation because these modes have been shown to sufficiently describe important contributions to global fluctuations necessary for protein function [21–24]. Flexible regions with important functional roles can be discriminated by considering fluctuations associated with correlated motion [25]. Information regarding coordinated motion for each residue can be obtained with the GNM from the cross-correlation matrix. While the definition we present here is conservative since important transitions known for protein function have been observed in other modes, it provides an initial training set that allows a support vector machine (SVM) to model the sequence subspace that encodes flexible regions with functional importance. Furthermore, with this approach we are able to identify FFRs using only a single protein conformation, making it possible to quickly generate the training dataset needed to build a prediction tool. Eliminating the need to extrapolate motion between two protein conformers allows us to expand the size of our training set.

Correlation values were used to weight mode information to create an FF score and empirically define a threshold to objectively identify FFRs (see Materials and Methods). The FF scores are then normalized such that the mean value is 0 and the standard deviation is 1 in order to establish a standard threshold for all proteins in the training set. The threshold is established based on the hypothesis that fluctuations of functional importance will deviate from the mean fluctuation observed for the entire protein. Therefore, we consider residues with a normalized FF score greater than 1.5 standard deviations from the mean fluctuation to exhibit flexibility of functional importance.

The FF score is used for definition purposes only. With this definition procedure we are able to obtain an objectively defined dataset needed for SVM training. The dominant motions in the lowest amplitude modes correspond to rigid domain motions [26,27]. The normalization procedure above, scaled with the correlation value, identified the extreme fluctuations within the rigid domains. Positive FF scores indicate large fluctuations relative to the intrinsic fluctuation state of the entire protein, whereas negative values indicate smaller than average fluctuations. Regions such as recognition and activation loops will fall on the extreme positive end of this FF score spectrum. Although low values of extracted GNM modes correspond to stable regions with negligible fluctuation, extreme negative FF scores correspond to hinge regions—the rigid domain fluctuations, modeled by the GNM, will be moving with respect to the hinge itself. Therefore, the hinge will appear to be immobile with the observed fluctuation falling below the overall mean fluctuation of the protein. Based on the examples provided subsequently, we show that this operational definition of FFRs sufficiently defines biologically confirmed flexible regions.

The FF score was first tested on HIV protease. While the recognition loop (residues 36 to 42) is identified without incorporating correlated movement information to weight normalized GNM fluctuations, the flap region (residues 46 to 56) important for dimerization was not identified because fluctuation is suppressed in the dimerized state (Figure 1). We subsequently show that incorporating information regarding correlated residue movements in a weighting scheme to rescale the GNM mode (see Materials and Methods) improved the identification of FFRs. The biological function of a protein is often achieved through coordinated movements; thus, the FF score uses values extracted from the cross-correlation matrix to weight residue participation in correlated fluctuations. Furthermore, fluctuations that are biologically important for protein function are often defined by their correlated nature [25]. As a result of this weighting scheme, residues with little participation in correlated movements are rescaled to have lower FF scores, whereas those with high correlation to other residues will have higher scores. Correlated and anticorrelated fluctuations are accounted for by summing the square of maximum and minimum correlation values, which are then used to scale the weighted average of the two slowest modes (see Materials and Methods). Using this weighting scheme in the FF score, we are able to improve our definition of FFRs. For HIV protease, the weighted FF score enabled us to detect the flap region and correctly categorize it to be functionally important (Figure 1C and 1D).

**Figure 1:**
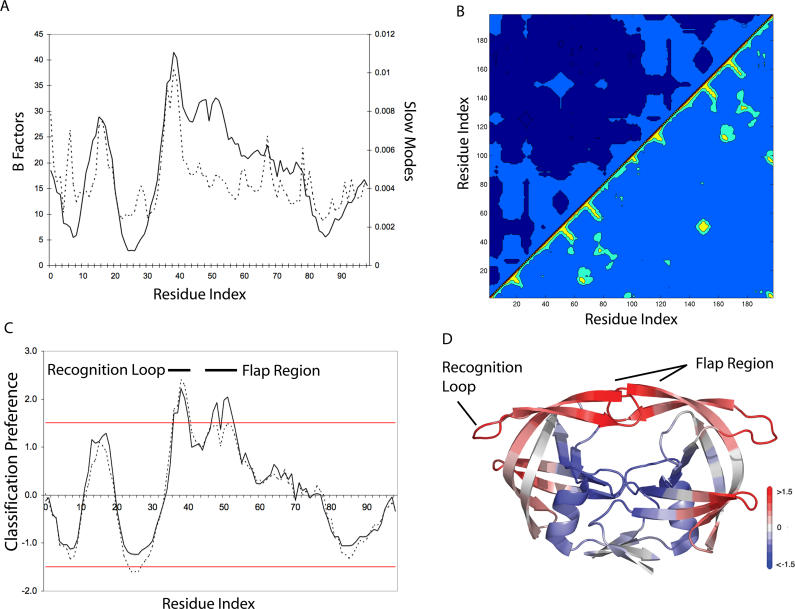
Defining FFRs in HIV Protease Using the Derived FF Score (A) Comparison of temperature factor (dashed line) and weighted average of the two slowest modes (solid line) obtained with GNM. The HIV protease is modeled as a dimer; however, the plot shows results for a single chain. (B) Gradient plot ranging from correlated (red) to anticorrelated (blue) movement for each residue in the dimer. (C) Comparison of normalized scores for unweighted (dashed line) and correlation-weighted (solid line) modes for a single chain. Correlation-weighted modes define the FF score. Regions are identified as FFR when values exceed thresholds (red lines) greater than 1.5 and less than −1.5. The flap region (residues 46 to 56) exceeds the threshold after including correlated movement information (solid line). (D) Structural mapping of FF score with gradient from negative (blue) to positive (red), (PDB ID: 1HIV).

Improvements in defining FFRs using the FF score were also observed for calmodulin and bovine pancreatic trypsin inhibitor (BPTI) (Figure 2). Calmodulin is a signaling protein consisting of an alpha helical hinge between two globular domains. While the two globular domains have been found to be structurally similar to each other, they differ dynamically [28–30]. These differences are also observed in the GNM modeling result that shows the N-terminal domain to be more flexible than the C-terminal domain. However, it is the interconnecting helix, containing eight turns, that has been observed to undergo the largest structural change upon calcium and substrate binding [31,32]. When bound to a peptide, a kink is introduced in this alpha helical hinge leading to a collapse that forms two perpendicular alpha helices while the globular domains wrap around the peptide. FF scores less than −1.5 identify this hinge region between the two globular domains despite having an ordered alpha helical structure.

**Figure 2:**
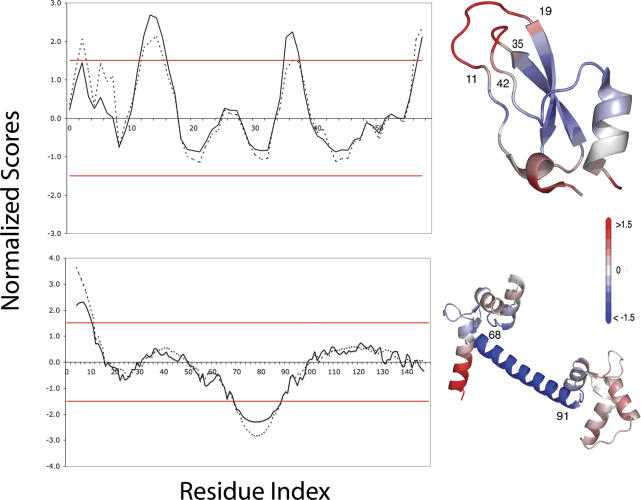
FF Score Identifies FFR in Bovine Pancreatic Inhibitor and Calmodulin Comparison of unweighted (dashed line) and weighted (solid line) FF scores for BPTI ([top], PDB ID: 5PTI) and calmodulin (bottom, PDB ID: 1CLL). FF scores are mapped with the same gradient coloring from negative (blue) to positive (red) as the scale shown in Figure 1D. Both recognition loops (loop 1: residues 11 to 19; loop 2: residues 35 to 42) are identified in BPTI by the FF score, whereas loop 2 is not identified with the unweighted mode. For calmodulin, the FF score allows us to identify the central hinge for this protein (residues 68 to 91 shown in blue because it exceeds the negative threshold of less than −1.5). This central helix, containing eight turns, is known to collapse when bound to calcium and substrate.

The binding affinity of BPTI is influenced by mutations in the active loops (residues 11 to 19 and 35 to 42) that are inserted into the active site of the proteolytic enzymes. Mutations Y35G [33] and G37A [34] lead to an observed increase in the fluctuation of these loops and reduce the binding affinity for trypsin compared to the native form. Structurally, the monomer G37A mutant adopts a near wild-type conformation based on comparison of nuclear Overhauser effects in NMR structures, whereas the structure of the Y35G mutant showed a 6-Å root-mean-square deviation from the native structure. Nevertheless, both mutant proteins showed a native conformation when bound to trypsin. In this example, we stress that while both proteins continue to adopt wild-type conformation according to experimental studies, their dynamics and stability varied substantially. The regions that were impacted the most by these mutations have been identified by our definition. Residues in these loop regions are defined to be FFRs with FF scores exceeding the threshold of 1.5. While this threshold is arbitrary and defined empirically, it provides a consistent definition which we can use as targets for training our predictors to identify sequence patterns that correspond to these regions.

### Features of FFRs

Based on the FF score, each residue in a nonredundant training set was classified as FFR or non-FFR. Residues were separated into a binary classification with FFRs assigned a value of 1 and non-FFRs were assigned a value of −1. Examining the distribution of residues in the two classes shows that an FFR averages 9 ± 11 residues in length and comprises about 20% of all residues. Residues identified as hinges comprise about 0.75% of all residues in the training set. The average maximum length of an FFR for each protein increases with increasing protein length (Figure 3A). This increase in length may be associated with longer flexible regions forming linker regions between multiple domains.

**Figure 3:**
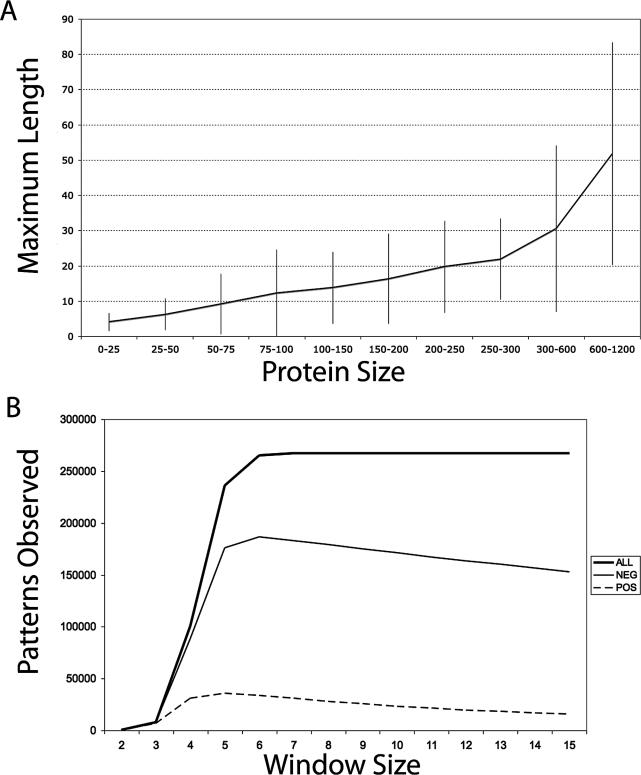
Preliminary Analysis of FFR as Identified by FF Score (A) The average of all maximal FFR lengths plotted against overall protein length. (B) The number of different sequence patterns observed for a given window size. Shown are the pattern counts for regions classified as FFR (dash line), non-FFR (thin line), and irrespective of classification (thick line). FFR regions sample a smaller sequence space compared to non-FFR regions. Patterns overlapping boundaries of FFR and non-FFR are excluded from these counts.

We examined the classification preference for each amino acid and secondary structure type using the same assignment values (1 and −1) (Table 1). Residues in beta strands generally make up protein cores and are less likely to constitute FFRs than helices or loops, a trend observed in the data (Table 1). Charged residues show stronger preferences to be in FFRs than non-FFRs (Table 2). Glycine was not among the top ranking residues, ranking even lower than proline. This is expected since the conformational flexibility and nonrestrictive properties of glycine make this residue very adaptable. Moreover, glycine is found both at the surface and in the hydrophobic core with no strong preference for either. Proline is known to be a helix breaker due to the conformational restraints of the covalent bond between the side chain and backbone. This conformational limitation means that proline is more likely to be found in loops and hence have a higher FF score. As expected, hydrophobic residues tend to be found in regions of less flexibility since they are packed into the hydrophobic core. Cysteines are frequently involved in disulfide formation and were found among the least common residues in FFRs. The large standard deviations found for these classification preference indicate that neither secondary structure nor amino acid residue properties alone are sufficient to serve as the distinguishing factor for classification of FFRs.

**Table 1:**
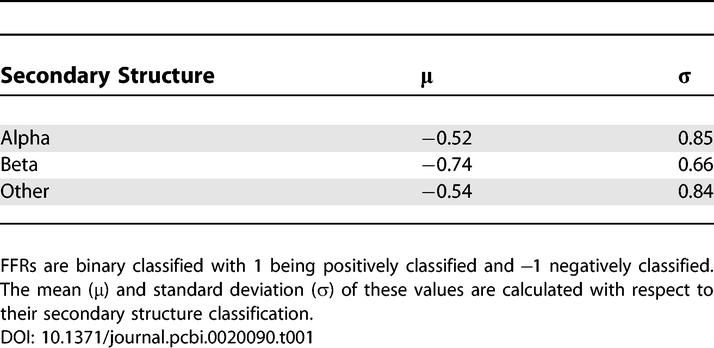
FFR Classification Preference for Secondary Structures

**Table 2:**
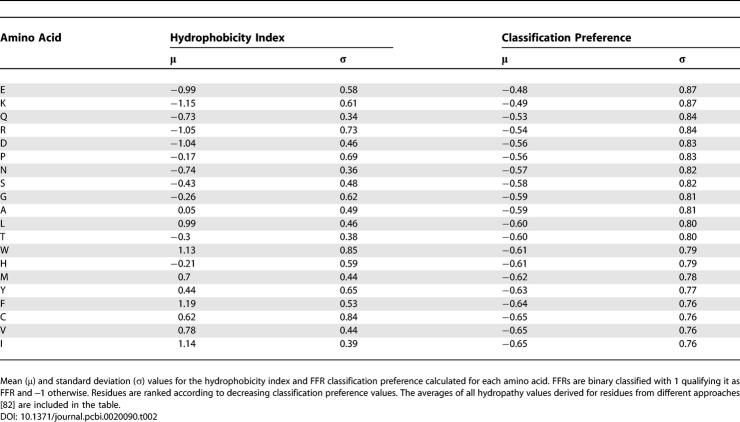
FFR Classification Preference for Amino Acids

### Accessing Sequence Pattern Preferences for FFRs

Window scanning for particular patterns reveals that FFRs occupy a smaller sequence space than their non-FFR counterparts (Figure 3B). For a window size of 2, all possible amino acid pairs are sampled by both FFRs and non-FFRs. The majority of triplets continue to be sampled for a window size of 3. Differences in pattern sampling become more evident for window sizes 4 and larger, indicating sequence preferences for FFRs and non-FFRs.

Certain tripeptide sequences can be overrepresented in FFRs when compared to non-FFRs. We attempt to identify these tripeptides by using a modified bootstrapping approach to calculate *Z-*scores and *p*-values for association with FFRs (see Materials and Methods). For a window size of 3, a total of 8,000 tripeptide sequence patterns are possible. There were 7,982 patterns observed in the training set, with 7,261 patterns in FFR regions and 7,967 in non-FFR regions. The modified bootstrap sampling with 10,000 repetitions for the respective subset size showed 429 patterns in the FFR pool to be statistically associated with that category using a *p*-value threshold of 0.05. These patterns are either underrepresented or overrepresented in FFRs compared to the null FFR model, making it a distinctive set to help identify these regions. While the statistical associations are weak, using these values as additional input features improved the prediction performance of SVMs.

Results from this analysis suggest that there are sequence patterns associated with these regions that may be detected using machine learning techniques and these findings have been instrumental in improving the prediction quality of our SVM-based predictors when incorporated. The rationale behind the modified bootstrapping was to identify tripeptide sequence patterns associated with FFRs and to use this information to help SVMs distinguish between FFRs and non-FFRs. This finding of context dependence supports previous work that has shown that the Flory isolated-pair hypothesis does not hold true [35]. This hypothesis states that the backbone conformation of residues is influenced by the nearest-neighbor residues rather than being independent of their conformations.

### SVM Architecture and Training

While many successful structure predictors use multiple sequence alignments or position specific scoring matrices, we chose to use hidden Markov models (HMMs) because they additionally capture insertion and deletion probabilities that may occur within the sequence [36,37]. As such these probabilities capture information regarding the conservation of sequence length that can be particularly important for identifying active sites or recognition loops limited to certain lengths. A total of 29 transition and match states were used as input features to the SVM (see Materials and Methods).

Exploring the performances of various SVM architectures have shown that a two-layered architecture yields the best performing predictor to identify residues in FFRs. The first-layer SVM makes an initial classification based on sequence and evolutionary information contained in the HMM states. The second-layer SVM serves to smooth the prediction from the first-layer SVM and uses results obtained from the modified bootstrap analysis to make better predictions. Incorporating information regarding tripeptide classification preferences was instrumental to improving the performance of our final predictor despite having a weak statistical value. Compared to a predictor that does not include tripeptide classification preferences, the performance of the SVM showed an additional 5% increase in accuracy and precision with an additional 3% improvement in recall.

The predictive performance of the SVMs was found to be a function of protein length. High false-positive rates were observed for shorter proteins (Figure 4A). This high error rate may be a result of original misclassification by the FF scores. For shorter protein segments, flexible regions are more likely to be assigned as non-FFRs because the dynamics of the segments will be modeled in a complex as opposed to a free monomer. Stated another way, it may be difficult to say whether these segments are intrinsically flexible in the apo form since they are always found with their binding partners. In total, complexed proteins compose 43.4% of the training set; 49.8% of proteins smaller than 200 residues are in complexes as compared to 35% found for larger proteins. Moreover, smaller proteins in crystal structures may be truncations or mimics of a flexible loop from a larger protein, leading to the misclassification of an FFR as a rigid segment even though the region may be flexible biologically.

**Figure 4:**
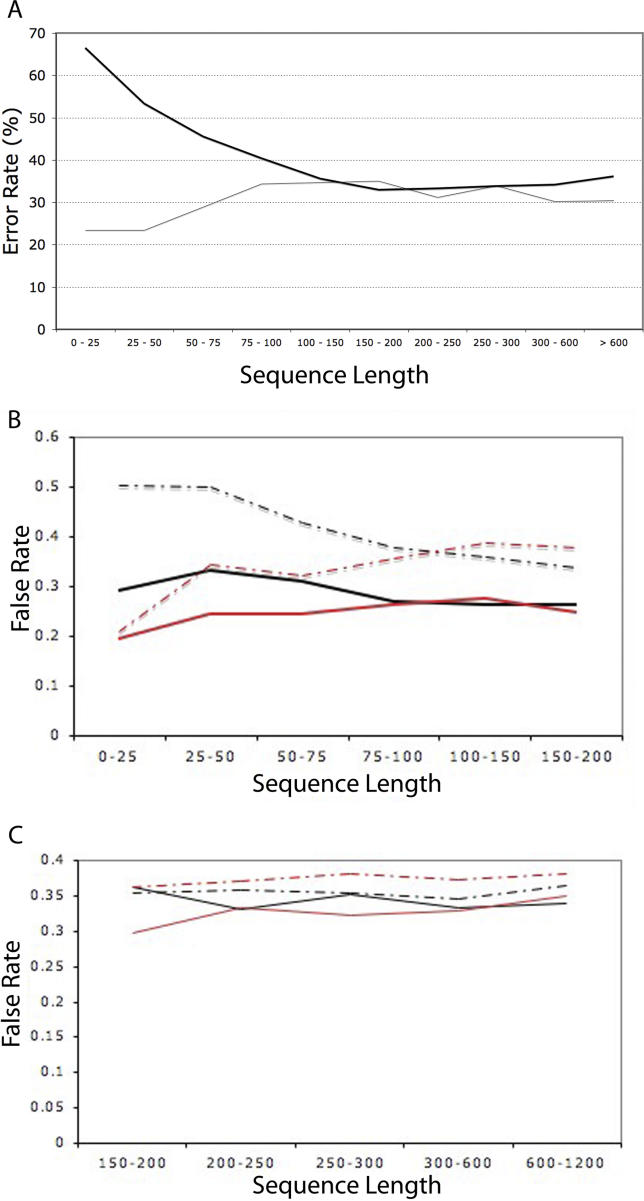
Predictor Performance Is a Function of Protein Length (A) Sequence effect on false-positive (thick line) and false-negative (thin line) error rate. Shorter sequences tend to have higher false positive identification of FFRs when trained on a nonpartitioned dataset. (B) Comparison of SVM prediction results trained on a nonpartitioned dataset (dashed lines) and a partitioned dataset containing proteins up to 200 residues (solid lines). Improvements were seen in both the false-positive (black) and -negative (red) rates. (C) Comparison of SVM prediction results trained on a nonpartitioned dataset (dashed lines) and a partitioned dataset containing proteins larger than 200 residues (solid lines). Minor improvements were observed in false-positive (black) and -negative (red) rates.

To account for protein length, the original training set was partitioned into two sets: A, 760 proteins up to 200 residues in length; and B, 574 proteins longer than 200 residues. SVMs trained on the partitioned training sets both showed an improvement in performance (Figure 4B). Training on subset A showed an overall improvement of 12% in recall and 7.8% in precision for a total accuracy of 76.46%, precision of 48.99%, and recall of 78.27%, whereas training on subset B showed only a slight improvement over training on all proteins (Figure 4C) with an accuracy of 66.01%, precision of 37.11%, and recall of 70.49%.

Our final predictors, Wiggle and Wiggle200, use the radial basis kernel function in the first layer and a linear kernel in the second layer. Wiggle is the product of training on all proteins and Wiggle200 was trained on subset A containing proteins up to 200 residues. Since minor improvements were observed for the predictor trained on the subset containing larger proteins, we use Wiggle to conduct our predictions. In the following discussion, we will first revisit the dependency of the predictors on protein size in regard to domain boundary detection. Then we will discuss the performance of the predictors on three examples with experimentally verified FFRs.

### Domain Boundary Identification

Flexible linkers between domains, sometimes acting as a hinge, are examples of FFRs and we evaluate the performance of Wiggle and Wiggle200 in the detection of these regions. We use a comprehensive domain boundary benchmark set (BENCH) that was curated to reflect the consensus of experts (CATH, SCOP, and authors of the protein structures) (T. Holland, S. Veretnik, I. N. Shindyalov, and P. E. Bourne, unpublished data). Because the boundary is defined between two residue positions, we expand the definition up to a window size of 15 residues, with the boundary in the center, to evaluate the performance of the predictors. We also partitioned BENCH based on protein size into BENCHA (200 residues or fewer) and BENCHB (more than 200 residues).

The general trend in predictor performance for Wiggle and Wiggle200 observed for all datasets (BENCH, BENCHA, BENCHB) is that precision increases with the size of domain boundary expansion, whereas recall increases up to window size 5 and begins to decline afterward (Figure 5). The overall accuracy is observed to decrease with a difference of about 2% for all datasets. For this reason, we will focus our performance comparison between the two predictors on a window size 5.

**Figure 5:**
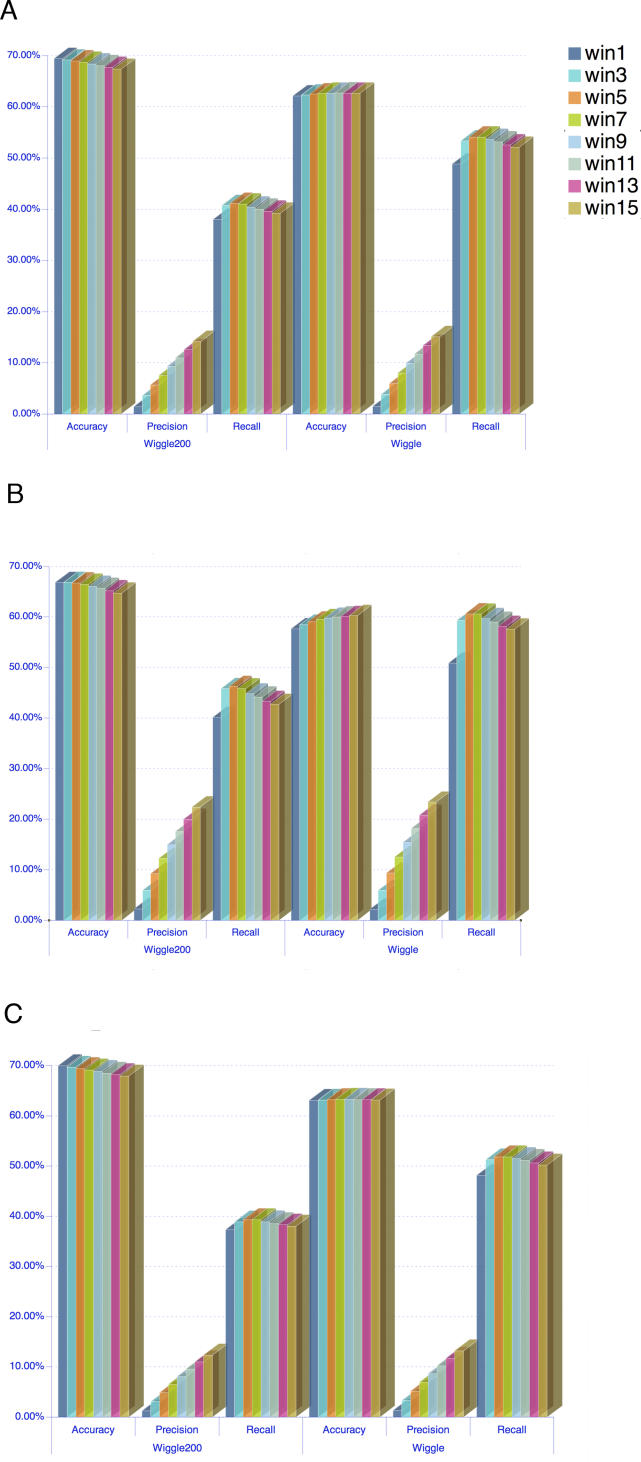
Predictor Performance in Identifying Domain Boundaries Wiggle predictors were evaluated for domain boundary predictions on (A) a benchmark dataset containing domain boundary consensus between experts (BENCH), (B) a partitioned BENCH with proteins up to and including 200 residues (BENCHA), and (C) a partitioned BENCH with proteins longer than 200 residues. Definitions of domain boundaries were expanded up to a window size of 15 (win15) with the boundary in the center.

For BENCH, we find that Wiggle outperforms Wiggle200 in recall by an additional +12.99% with little improvement in precision (+0.31%) and a decrease in accuracy (−6.44%). Wiggle identifies domain boundaries in BENCH at an accuracy of 62.55% with a precision of 6% and recall of 54.15%. We are not surprised to see a poor precision value since both predictors will identify other flexible regions that are not linkers between domains. However, the results here show that our predictors are identifying linkers between domain boundaries, for example, possibly serving a functional purpose as a hinge.

For the partitioned benchmark set (BENCHA and BENCHB), we find that Wiggle again outperforms Wiggle200 in domain boundary recall with an additional +14.34% and +12.51%, respectively. Again, minor improvements were observed in precision (BENCHA: +0.13%, BENCHB: +0.31%) and a slight decrease in accuracy (BENCHA: −7.68%, BENCHB: −6.19%) was observed for Wiggle compared to Wiggle200. For the partitioned datasets, BENCHA and BENCHB, Wiggle predicts boundaries at (BENCHA: 59.08%; BENCHB: 63.24%) accuracy, (BENCHA: 9.39%; BENCHB: 5.21%) precision, and (BENCHA: 60.66%; BENCHB: 51.85%) recall, respectively. This clearly indicates that Wiggle, trained on the entire training dataset which includes larger multidomain proteins, has picked up sequence patterns associated with linker regions and is the better predictor for domain boundaries compared to Wiggle200.

### SVM Performance on Experimentally Verified FFRs

Although the GNM provides a fast approach to identifying FFRs, there are limitations to the model. Dynamic modeling results are largely dependent on protein conformation, particularly that defined by bound and unbound conformations as discussed earlier for the observed higher false-positive error rate for smaller proteins. Therefore, the FF score does not always correctly define the regions of interest. We examined a few case studies where residues were largely misclassified by the FF score and compared the results to our SVM predictions. While it is ideal to have a precisely classified training dataset, we concluded that the classification made by the FF score provides a sufficient training set for the SVM to detect correct signals in sequence patterns for FFRs. In short, SVMs are powerful enough to generalize the relationship between protein sequence and FFRs as illustrated in the following examples.

#### Arc repressor.

The arc repressor is stable as a dimer, unfolded as a monomer [38–41], and bound to DNA as a tetramer [41,42]. Extensive mutagenesis has been conducted to identify residue contributions to activity and stability [43]. The beta strand near the N-terminus, the site of DNA interaction, is the least tolerant to substitution when selected for activity, but mutations have minimal effects when selected for stability. The loop between the two alpha helices (residues 28 to 34) was found to be intolerant to substitution under both circumstances. Based on these mutagenesis studies, these are some of the target regions we wish to identify using our sequence-based predictors.

Structurally, several flexible regions having important roles for protein function have been detected in the arc repressor using various experimental techniques. Despite being highly disordered in solution, according to an NMR structure determination [44], the N-terminus of the repressor (residues 1 to 9) is important for specific operator binding [45]. The last three residues of the C-terminus are also found to be disordered in solution [44], while remaining residues at this terminus have been found to contain important contacts for tetramerization [46]. Hydrogen exchange experiments show the exchange rates for the two alpha helices are concentration dependent and suggest that the protein exists as a molten globule in a monomeric state [38]. In order to make all the DNA contacts observed in operator binding [47], four molecules of arc repressors are needed, suggesting the existence of a tetrameric state. To shift from a monomeric to dimeric and finally tetrameric state requires considerable accommodation for conformational change. The flexibility of this protein required to accommodate these domain arrangements is not evident from the crystallographic or NMR structures alone.

Wiggle identifies residues 5 to 8, 23 to 35, 38, and 40 to 53 as FFRs, and Wiggle200 identifies residues 5, 23 to 29, and 43 to 53. The FF score only identifies residues 45 to 53 located at the C-terminus (Figure 6A). Residues 5 to 8, identified by Wiggle, correspond to the residues experimentally defined as important for DNA recognition at the N-terminus, while residues 23 to 35 and 38 correspond to the substitution-intolerant loop linking the two alpha helices [43,46] (Figure 6B).

**Figure 6:**
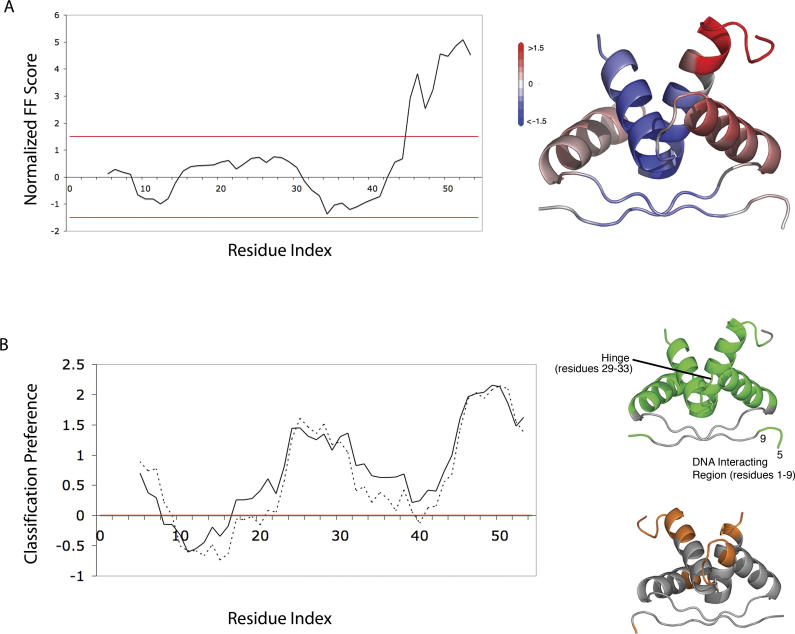
Performance of Wiggle Predictors on Arc Repressor (A) The dimer conformation of the Arc repressor was used to model global fluctuation. Using the FFR definition, the plot for a single chain is shown on the left with structural mapping of values onto a dimer on the right. FF scores are mapped with the gradient code from negative (blue) to positive (red). Only the C-terminal tail exceeds threshold lines (red) and is defined as an FFR while the rest of the protein is not. (PDB ID: 1BAZ) (B) The hinge between the two helices is identified by predictors as well as N-terminal residues important for DNA recognition. Predictions from Wiggle (solid line) are mapped in green on the structure and Wiggle200 (dashed line) are mapped in orange.

#### PVUII endonuclease.

PVUII endonucleases (156 amino acids) are homodimerizing proteins that catalyze highly specific DNA cleavage. No regions of flexibility were identified with the FF score (Figure 7A). Wiggle identified residues 2 to 10, 26 to 31, 33 to 38, 65 to 68, 116 to 118, 121, 132 to 138, and 146 to 157 as FFRs, and Wiggle200 identified residues 2 to 8, 33, 34, 36, 53 to 58, 60, 61, 94 to 96, 117 to 120, and 150 to 157. Both predictors identified the loop involved in minor groove recognition (residues 26 to 36) [48], Mg^++^ ion coordination (residues 58, 67, 68, 82, and 94) [49], and catalytic activity (residue 34) [48,50] (Figure 7B).

**Figure 7:**
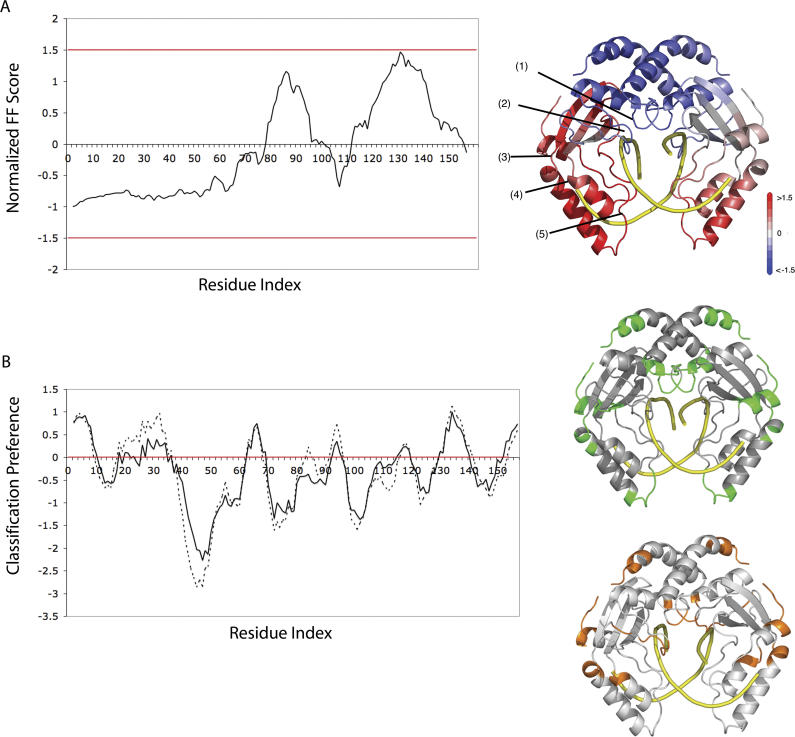
Wiggle Predictors Identify Important FFR in PVUII Endonuclease (A) Plot of FF scores and mapping of values in a gradient code from negative (blue) to positive (red) onto the structure of PVUII endonuclease in complex with DNA (yellow). The following structural features are labeled: (1) minor groove binding loop, (2) catalytic loop, (3) potential hinge for DNA binding, (4) tyrosine 94 for Mg^++^ ion coordination, and (5) major groove binding loop. (PDB ID: 3PVI). (B) Wiggle predictions (solid line) are mapped in green and Wiggle200 predictions (dashed line) are mapped in orange onto the structure.

Y94 coordinates Mg^++^ ions needed for endonuclease activity in this restriction enzyme [49]. Despite the availability of numerous crystal structures for this protein, no electron density was observed for Y94 until the enzyme was cocrystallized with Mg^++^ [49] ion, a necessary cofactor for protein function. This is indicative of the need for FF to facilitate metal ion binding, a result supported here. Structural inspection suggests that the other identified residues, unconfirmed in the literature, fall into regions that may serve as hinges for the major groove DNA recognition domain. This region could serve as a possible target for experimental studies to understand the dynamics of this protein.

#### Erythropoietin.

FFRs identified in erythropoietin contain examples where local flexible regions are stabilized by mutations or glycosylations, both of which are sequence modifications that result in a shift from a disordered to ordered state. No regions of flexibility were identified using the FF score (Figure 8A) in this protein which functions in initiating differentiation and proliferation of progenitor cells into red blood cells. The system modeled by the GNM is a bound unit of erythropoietin to the corresponding receptor (not displayed in Figure 8). As a result, the fluctuation of erythropoietin appears to be diminished.

**Figure 8:**
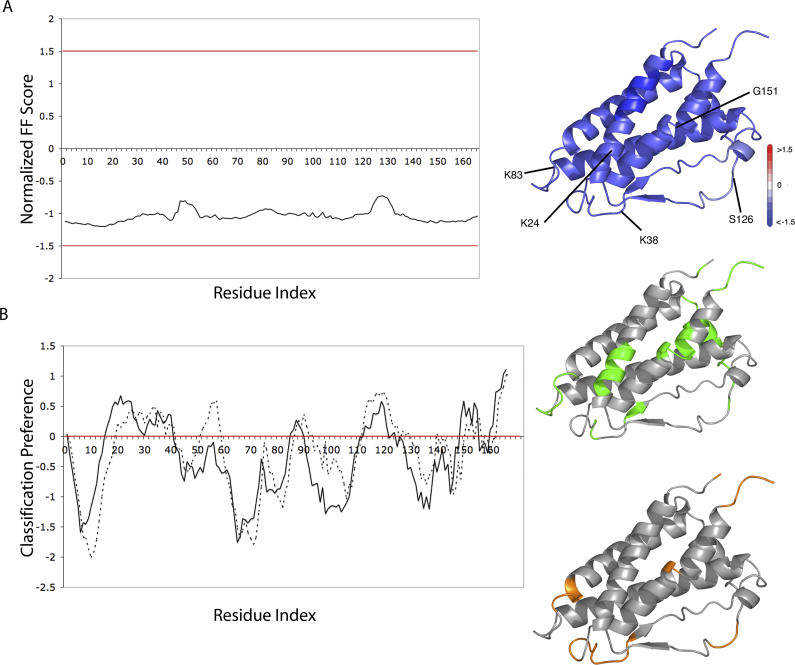
Wiggle Predictors Identify Regions Corresponding to Glycosylation Sites on Erythropoietin (A) FF score plotted against residue number with thresholds shown in red. Erythropoietin is modeled by the GNM in the complexed form with the corresponding receptor (not shown). All residues have below mean fluctuation (colored blue), but none of the residues are defined as FFRs since they do not exceed the definition threshold. The four glycosylation sites (S126 and lysine substituted K24, K38, and K83) along with G151 are labeled. (PDB ID: 1EER) (B) FFRs correspond to positive values as predicted by Wiggle (solid line) and Wiggle200 (dashed line) which are structurally mapped onto erythropoetin (green and orange, respectively). Not all loops are identified by the predictors to be functionally flexible, thus showing that discrimination is not based on structural features.

Overlaps were found between prediction results (Wiggle: residues 1, 16 to 40, 85 to 89, 113 to 121, 123, 124, 149 to 155, and 160 to 166; Wiggle200: residues 19 to 40, 50 to 57, 86 to 90, 92, 111 to 124, 126 to 128, 139, 150 to 152, 154, 155, 157, and 162 to 166) and correspond to mutations introduced for the creation of a soluble analog [51] to obtain a crystal structure. All five mutations (N24K, N38K, N83K, P121N, and P122S) reside in, or are immediately adjacent to, positively classified regions (Figure 8B). These mutations include lysine substitutions made at *N*-linked glycosylation sites and prolines removed from the CD loop which contained conformational heterogeneity. Wiggle identified the CD loop and all glycosylation sites as FFRs, with the exception of one glycosylation site where the adjacent region is predicted. Additionally, a kink introduced by G151 was also identified as an FFR.

Glycosylation of erythropoietin is necessary for its biosynthesis and bioactivity and plays a critical role in its stability [52,53]. Removal of all carbohydrates results in aggregation of the protein [54] and can be made soluble in vitro with mutations N24L, N38L, and N83L [14]. However, these mutations do not prevent the formation of insoluble aggregates or rapid degradation in vivo [52]. Carbohydrates increase the half-life of this hormone, but binding affinity is negatively impacted by 20-fold [55].

G151 plays an important structural role by introducing a kink in the αD helix. This enables K152 to come in contact with residues in the protein core to form one of the two interaction sites for erythropoietin receptors. Alanine replacement in either position 151 or 152 resulted in a substantial loss of bioactivity [56]. Both of these positions were identified by Wiggle predictors as FFRs. Binding of erythropoietin to its receptor leads to a slight increase in alpha helical content [57]. NMR and X-ray structures of erythropoietin in the unbound and bound state, respectively, showed the formation of a small alpha helix occurring in the highly flexible CD loop and a less pronounced kink observed at G151 in the receptor bound X-ray structure [58]. These are examples of two regions where the structural changes resulting from binding interactions to the receptor correspond to local flexible regions allowing these changes to occur.

Mutagenesis performed to identify erythropoietin receptor binding sites revealed four regions (residues 11 to 15, 44 to 51, 100 to 108, and 147 to 151) important for the activation of receptor signaling [59]. With the exception of residues 149 to 152, functionally flexible predictions were made outside of these binding hot spots. This shows that our predictors are not trained to predict binding sites, but rather regions where flexibility is important for bioactivity or accommodating different conformational states.

### Comparison to Protein Disorder Predictions

Several protein disorder predictors were compared to Wiggle and Wiggle200 predictions (Figure 9) to illustrate that these predictors identify different targets. Disorder predictors differ widely in their approaches, but targets are generally based on high temperature factors or missing residues in crystal structures. PONDR [60] is a disorder predictor trained on fractional composition and hydropathy. DISOPRED [61] uses the PSI-blast matrix as input to an SVM to detect disorder, while DisEMBL [62] is a neural network trained for the predictions of coils, hot coils, and disorder. RONN [63] uses a bio-basis function neural network to take advantage of information embedded in homologous proteins. GlobPlot [64] and FoldIndex [65] are simpler algorithms that, respectively, use running propensity for protein disorder and an index that classifies residues based on hydrophobicity and net charge. IUPRED [66] uses concepts of pair-wise interaction potentials observed in globular proteins to make assignments for each residue. Finally, NORSP [67] assesses regions based on low confidence predictions for secondary structural elements.

**Figure 9:**
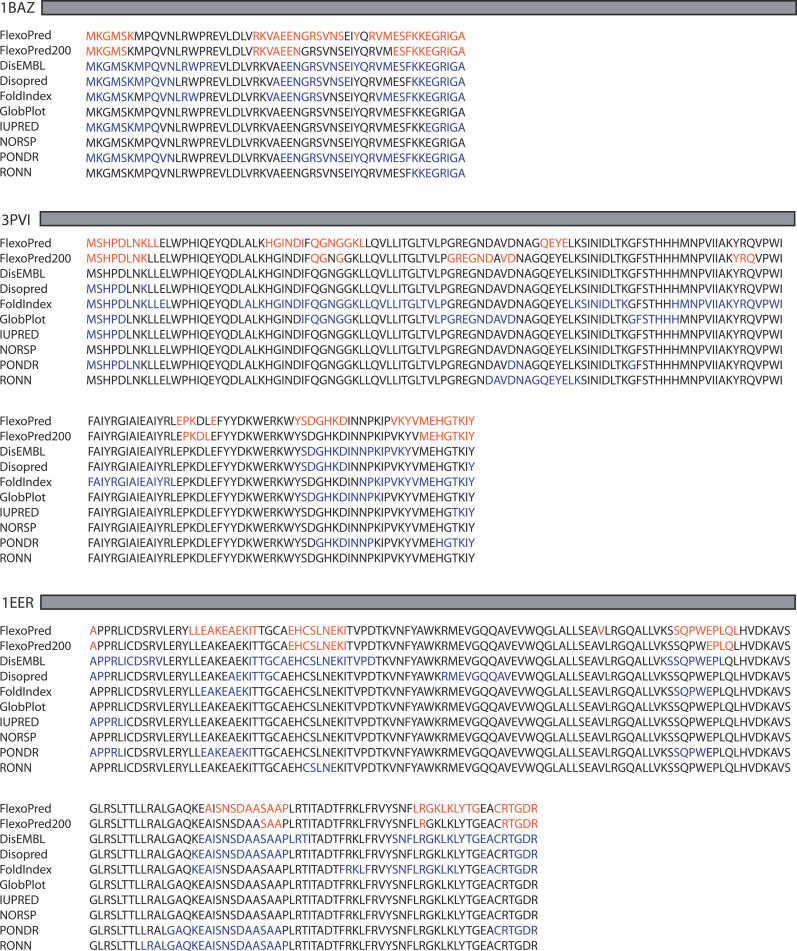
Comparison of Wiggle Predictors to Structural Disorder Predictors Comparison of prediction results from Wiggle (red) to various disorder predictors (blue).

Some overlaps are expected with disorder predictions because FFRs may be disordered depending on the conformational state of the protein. Otherwise, we expect little correlation since disorder predictors generally aim to identify structural disorder and regions with a low propensity to form an ordered unit. Potential functional roles were not considered in their design, although these regions are suggested to be important for protein-protein recognition after examining positively classified sequences [68,69]. With the exception of the arc repressor where predictor results exhibited significant overlap, Wiggle and Wiggle200 have been found to target regions that were not otherwise identified by disorder predictors.

For arc repressor (1BAZ), disorder predictors positively classified terminal ends, although some failed to identify it altogether. The hinge region connecting the two helices is not fully identified by most disorder predictors. While Wiggle predictors did not identify all residues involved in recognition at the major groove for PVUII endonuclease (3PVI), it identified the minor groove recognition loop, catalytic loop, and magnesium ion coordinating residues. Current disorder predicting tools failed to identify these regions. Disorder predictors that successfully identified at least one of these regions are based on an index separating hydrophobicity and net charge (FoldIndex and GlobPlot) or the use of homology information (RONN).

Most disorder predictors failed to identify all glycosylation sites on erythropoietin (1EER) with the exception of DisEMBL, having the most overlap in predictions with Wiggle. The structure of erythropoietin is entirely helical, and DisEMBL has been designed to predict coils with high B factors. The glycine kink was also missed by most disorder predictors except for DisEMBL and FoldIndex.

We also compare the performance of predictors in identifying FFRs as defined by the FF score (Table 3). Two test sets were used: TESTALL and TEST200 containing randomly selected chains from the training dataset for all proteins and proteins up to 200 residues long, respectively. These test sets were used during one of the cross-validation runs from which the Wiggle predictors were created; therefore, the performance results reflect unseen cases for Wiggle. The results show that DISOPRED was able to identify FFRs with the highest accuracy for both test sets (TESTALL: 78.48%, TEST200: 75.20%). However, DISOPRED failed to identify FFRs as indicated by the poor recall (TESTALL: 11.54%, TEST200: 12.89%). The predictor is therefore poor at identifying FFRs by identifying most residues to be a non-FFRs despite having a high precision. We observed earlier that the residue pool is disproportionate with the FF score identifying about 20% of the residues to be located in an FFR.

**Table 3:**
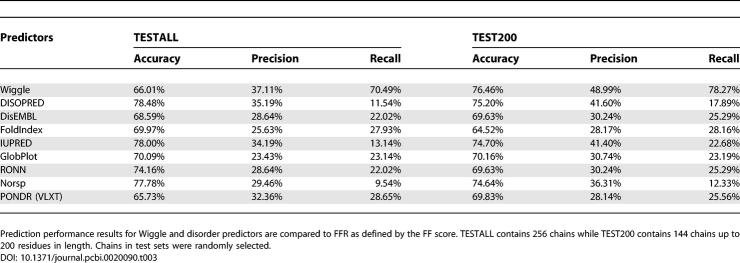
Comparison of Predictors Using TEST200 and TESTALL

We report the performance of Wiggle on TESTALL and Wiggle200 on TEST200. Wiggle predictors outperformed the other disorder predictors in overall performance for both test sets when comparing precision and recall values (Table 3). These results are expected since the predictors were all trained to identify a different target property of proteins. Our predictors were designed to identify regions of flexibility with functional importance unlike the other predictors that target highly disordered regions. The comparison of predictors is an important demonstration to illustrate that the target regions identified are different. This comparison is not intended to measure or make an assessment regarding the ability of Wiggle predictors to identify protein disorder. That our test cases are actually solved structures implies some level of order for the regions to be identified.

### Conclusion

The motivation for this work is to advance our understanding of protein sequence and FF through easily applied in silico methods. Protein fold and disorder properties are encoded in the amino acid sequence. We believe that functionally important protein flexibility is also encoded in the primary sequence and have successfully created tools to identify these regions. We created two predictors; one specialized for proteins shorter than 200 residues and another for all proteins regardless of size. Between the two predictors, we correctly identified flexible regions of functional importance in several test cases where structure-based classification had difficulties. Our targets include hinges, recognition loops, and localized regions that may serve to accommodate entropy dislocation necessary for allostery.

We focused on regional motion important for protein function based on residue participation in correlated low-frequency fluctuations that correspond to large global changes as modeled by the GNM. Our predictors differ from other predictors by including an additional functional consideration in our targets used for training our SVMs. Secondary structure predictors are trained against well-ordered regions of proteins to identify regular secondary structural elements and disorder predictors have been trained using various definitions that include regions missing electron density in X-ray structures or have high temperature factors. Both focus on a subset of sequence space important for structural features but do not address patterns involved in modulated protein flexibility that switch between ordered and disordered states.

With the Wiggle predictors, we were able to show detection of domain boundary and experimentally confirmed FFR in specific examples. Comparison to disorder predictors shows that, while there are expected overlaps, different regions are identified. The difference between predictors is that Wiggle predictors are trained to select for residues participating in the two largest modes of global motion, whereas disorder predictors were trained on the propensity to form ordered structures or lack thereof.

While false prediction error rates are approximately 30%, this may largely be attributed to the difficulties of defining our regions of interest with misclassifications occurring in both directions when using the FF score. SVMs trained on partitioned datasets showed improved performance, suggesting that the characteristics of FFRs are related to protein size. The Wiggle predictors are especially useful for proteins where no structural data are available. Localizing regions of FF in the absence of structural information will help identify mutational hot spots that may modulate bioactivity and these regions can be targeted in protein engineering experiments. The identification of FFRs by sequence-based methods complements and reduces the limitations in structure-based definitions of flexible regions.

## Materials and Methods

### Training set.

A nonredundant training set of protein chains with percent sequence identity of less than or equal to 10%, resolution better than 2.0 Å, and an R-factor less than 0.30 were retrieved from the PDB [70] using PISCES [71]. We further ensure nonredundancy by checking for distant protein homologs within the retrieved dataset using PSI-BLAST [72]. Each protein in the dataset was used as a query to search against a sequence database clustered with CD-HIT [73–75] at 90% identity. Distant homologs within the dataset (111 pairs) were eliminated if the sequence was retrieved by PSI-BLAST.

The final training set contained 1,277 sequences with 56.6% of the chains existing in the monomeric state. Multiple copies of a protein found in the asymmetric unit were eliminated. Complexes were manually inspected using the protein quaternary structure file server (PQS) [76] and literature confirmation sought for biological relevance. If the complexes were not found in nature, they were removed from the training dataset. The training set was then partitioned into two subsets based on protein length and used to train specialized SVMs. Subset A contained 720 proteins of length less than or equal to 200 amino acids; subset B contained 557 proteins of length greater than 200 amino acids.

### HMMs.

SAM-2tk [37] was used to build HMMs for all sequences in the training datasets. Homologs for each sequence in the training set were retrieved from a sequence database clustered at 65% identity with CD-HIT [73]. Clustering affects the probability states in the HMM; it was therefore important to check that patterns detected by prediction methods were not eliminated as a result. We tested the impact of CD-HIT on secondary structure predictions and found slight improvements in prediction quality (data not shown). Therefore, for reasons of increased remote homolog detection, reduced computational search time, and improved secondary structure prediction, the clustered sequence database was used in building HMMs using a target entropy weighting of 1.0 bit per column.

### GNM.

The GNM [16,77] combines the simplicity of the elastic theory applied to random polymer network [78] and the success of using a single-parameter potential [79] to model protein dynamics based on coordinates of the C_α_ atoms serving as nodes. The connectivity within the protein structure is represented as a Kirchhoff matrix Γ where R is the distance between the C_α_ atoms of residues *i* and *j* with r_c_ denoting the distance radius threshold (7 Å).


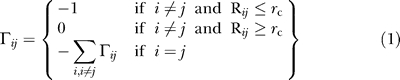


The equilibrium-correlated fluctuations between two sites can be obtained by finding the inverse of the Kirchhoff matrix and is represented as:

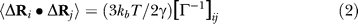
where *k_b_* is the Boltzmann constant, *T* is the absolute temperature, and γ is a single-parameter harmonic potential that accounts for the fluctuations of a residue about a mean axis.

Cross-correlated fluctuations between residues *i* and *j* are defined as:


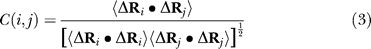


Participation in correlated movements was used to define flexible regions that are functionally important. Readers are referred to the original papers for details.

### Definition of FFRs.

Operationally, FFRs are defined using normalized FF scores. For each residue *i,* the maximum and minimum values, corresponding to residues *m* and *n,* respectively, are extracted from the cross-correlation matrix *C.* These values, *C(i,m)* and *C(i,n),* are used to scale the weighted average of the top two modes *j* of protein fluctuation where μ is the eigenmode and λ is the corresponding eigenvalue.


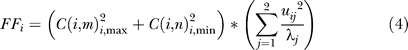


FF scores are normalized for each protein after removing outliers using a median-based approach [80]. To distinguish outliers, the median of the absolute difference *(mad),* taken between FF scores and the median of FF scores *(m_1_),* for the protein is first calculated. Each residue is then assigned an *M* value to identify and exclude outliers, defined by *M* > 3.5 and *M* < −3.5, prior to the calculation of the mean and standard deviation for normalization. For large sample sizes, the expected value of *mad* is 0.6745σ.

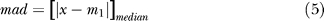


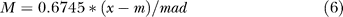
The calculated mean and standard deviation, obtained after exclusion of outliers, were used to normalize FF scores to a mean of 0 and standard deviation of 1. This normalization process rescales the protein fluctuation such that the mean fluctuation values are centered about the value 0.


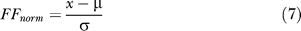


FFRs are defined to contain amino acids with *FF_norm_* > 1.5 or *FF_norm_* < −1.5. This threshold is chosen empirically based on the assumption that fluctuations differing from the mean fluctuation of the entire modeled system will be important for protein functionality.

### Bootstrapping for sequence preferences.

A modified bootstrap approach was used to identify sequence preferences for FFRs defined by the FF score. The aim of this analysis is to use these findings as additional input features for SVM-based classification. Protein sequences in the dataset were window scanned to pool triplets found in the training set. These pooled triplets were analyzed to identify sequence pattern distributions most correlated with FFR and non-FFR classifications. Two null models were created, one for FFRs and another for non-FFRs, by randomly selecting from the pooled triplets with replacement. Samples were drawn to be the same size as observed for FFR and non-FFR classes. *Z*-scores and *p*-values were calculated using the generated null model distribution for each observed triplet in their respective category. These classification preferences were included as additional input features to help the SVMs identify FFRs.

### SVMs.

All training schemes were performed with 5-fold cross-validation using SVM*light* [81]. Positively categorized residues were matched by one randomly selected negative residue to create a 1:1 ratio during training. The linear kernel model was initially used to conduct performance comparisons between different SVM architectures. This kernel was chosen because the need for parameter optimization is eliminated, thus providing a faster alternative for preliminary comparisons. Performances of SVMs were evaluated based on accuracy, precision, and recall where the ratio of relative true-positive (TP), true-negative (TN), false-positive (FP), and false-negative (FN) is examined. Unlike the training phase, no residues were excluded during performance evaluations of SVM performance.


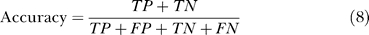



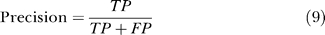



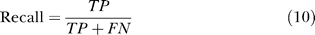


The predictor architecture for both Wiggle and Wiggle200 contains two layers. Input features for the first layer SVM include the nine HMM transition states and 20 match states. In HMM models, the match state probabilities give the probability of observing an amino acid at a particular position. The transition state probability is the probability of changing from one state (deletion, insertion, or match) to another from the previous state. For a window size of 9, a total of 261 (9 × 29) input features were used for each residue. Values are set to 0 when the window extends beyond terminal ends.

The prediction results from this first layer SVM is then included along with calculated *Z*-scores and *p*-values obtained for triplets from the modified bootstrap analysis as input features into a second-layer SVM. We find that using the radial basis kernel function to model input features for the first-layer SVM (γ = 0.25, C = 2) and the linear kernel function for the second-layer SVM to yield the best performing predictors.

With this two-layer architecture and optimized parameters, two different predictors were developed defined by their training sets. Wiggle was trained on the entire training set, while Wiggle200 is a more specialized predictor trained on proteins up to 200 amino acids in length.

### Assessment of domain boundary predictions.

Wiggle prediction results were compared to a benchmark dataset (BENCH) reflecting the consensus of domain boundaries among CATH, SCOP, and authors of the three-dimensional structures (T. Holland, S. Veretnik, I. N. Shindyalov, and P. E. Bourne, unpublished data).

This dataset contains 312 chains, of which 66% are multidomain proteins, covering 30 distinct architectures and 211 distinct topologies as defined by CATH.

The prediction performance was measured based on accuracy, precision, and recall values. Domain boundaries in the dataset were defined between two adjacent positions. We therefore investigated the performance of predictors for a variety of window sizes, up to 15 residues, with the boundary resting in the middle of the expanse. Performance evaluations were also tested on a partitioned benchmark set based on protein sizes up to 200 residues (BENCHA) and longer (BENCHB).

### Comparison of disorder predictors.

To compare residue classification of Wiggle predictors to different disorder predictors for the three specific protein comparisons, we set VSL1 version of PONDR to predict with a 10% false-positive rate, and DisEMBL to predict hot coils defined as coils with high B factors. Recommended defaults for a window size of 9 when requested were used for remaining predictors.

We also compare the performances of disorder predictors with two different test sets (TEST200 and TESTALL) containing randomly selected chains used during the training of Wiggle predictors. TEST200 contains 144 chains up to 200 residues and TESTALL contains 256 chains regardless of length. For disorder predictors, we used the same default values and settings as the specific case example comparisons with the exception of PONDR. The default predictor for PONDR (VLXT) was used to accommodate larger proteins in the test sets. Wiggle was used for TESTALL and Wiggle200 for TEST200.

## Abbreviations

BPTI: bovine pancreatic trypsin inhibitor
FF: functional flexibility
FFR: functionally flexible region
GNM: Gaussian network model
HMM: hidden Markov model
MD: molecular dynamic
SVM: support vector machine

## Note Added in Proof

The reference cited in the text as (T. Holland, S. Veretnik, I. N. Shindyalov, and P. E. Bourne, unpublished data) is now in press:

Holland TA, Veretnik S, Shindyalov IN, Bourne PE (2006) Partitioning proteins structures into domains: Why is it so difficult? J Mol Biol. In press.

## References

[pcbi-0020090-b001] Doruker P, Jernigan RL (2003). Functional motions can be extracted from on-lattice construction of protein structures. Proteins.

[pcbi-0020090-b002] Lu MY, Ma JP (2005). The role of shape in determining molecular motions. Biophys J.

[pcbi-0020090-b003] Gunasekaran K, Ma B, Nussinov R (2004). Is allostery an intrinsic property of all dynamic proteins?. Proteins.

[pcbi-0020090-b004] Kern D, Zuiderweg ER (2003). The role of dynamics in allosteric regulation. Curr Opin Struct Biol.

[pcbi-0020090-b005] Daniel RM, Dunn RV, Finney JL, Smith JC (2003). The role of dynamics in enzyme activity. Annu Rev Biophys Biomol Struct.

[pcbi-0020090-b006] Cooper A, Dryden DT (1984). Allostery without conformational change. A plausible model. Eur Biophys J.

[pcbi-0020090-b007] Post CB, Dobson CM, Karplus M (1989). A molecular-dynamics analysis of protein structural elements. Proteins.

[pcbi-0020090-b008] Whitten ST, Garcia-Moreno EB, Hilser VJ (2005). Local conformational fluctuations can modulate the coupling between proton binding and global structural transitions in proteins. Proc Natl Acad Sci U S A.

[pcbi-0020090-b009] Vergani B, Kintrup M, Hillen W, Lami H, Piemont E (2000). Backbone dynamics of Tet repressor alpha8intersectionalpha9 loop. Biochemistry.

[pcbi-0020090-b010] Muller CW, Schlauderer GJ, Reinstein J, Schulz GE (1996). Adenylate kinase motions during catalysis: An energetic counterweight balancing substrate binding. Structure.

[pcbi-0020090-b011] Clarkson MW, Lee AL (2004). Long-range dynamic effects of point mutations propagate through side chains in the serine protease inhibitor eglin c. Biochemistry.

[pcbi-0020090-b012] Todd AE, Marsden RL, Thornton JM, Orengo CA (2005). Progress of structural genomics initiatives: An analysis of solved target structures. J Mol Biol.

[pcbi-0020090-b013] Vucetic S, Brown CJ, Dunker AK, Obradovic Z (2003). Flavors of protein disorder. Proteins.

[pcbi-0020090-b014] Narhi LO, Arakawa T, Aoki K, Wen J, Elliott S (2001). Asn to Lys mutations at three sites which are N-glycosylated in the mammalian protein decrease the aggregation of Escherichia coli-derived erythropoietin. Prot Eng.

[pcbi-0020090-b015] Kuhlman B, Dantas G, Ireton GC, Varani G, Stoddard BL (2003). Design of a novel globular protein fold with atomic-level accuracy. Science.

[pcbi-0020090-b016] Bahar I, Atilgan AR, Erman B (1997). Direct evaluation of thermal fluctuations in proteins using a single-parameter harmonic potential. Fold Des.

[pcbi-0020090-b017] Micheletti C, Carloni P, Maritan A (2004). Accurate and efficient description of protein vibrational dynamics: Comparing molecular dynamics and Gaussian models. Proteins.

[pcbi-0020090-b018] Atilgan AR, Durell SR, Jernigan RL, Demirel MC, Keskin O (2001). Anisotropy of fluctuation dynamics of proteins with an elastic network model. Biophys J.

[pcbi-0020090-b019] Tozzini V (2005). Coarse-grained models for proteins. Curr Opin Struct Biol.

[pcbi-0020090-b020] Doruker P, Atilgan AR, Bahar I (2000). Dynamics of proteins predicted by molecular dynamics simulations and analytical approaches: Application to alpha-amylase inhibitor. Proteins.

[pcbi-0020090-b021] Temiz NA, Meirovitch E, Bahar I (2004). Escherichia coli adenylate kinase dynamics: comparison of elastic network model modes with mode-coupling (15)N-NMR relaxation data. Proteins.

[pcbi-0020090-b022] Chau PL, van Aalten DMF, Bywater RP, Findlay JBC (1999). Functional concerted motions in the bovine serum retinol-binding protein. J Comput Aided Mol Des.

[pcbi-0020090-b023] Hayward S, Kitao A, Berendsen HJC (1997). Model-free methods of analyzing domain motions in proteins from simulation: A comparison of normal mode analysis and molecular dynamics simulation of lysozyme. Proteins.

[pcbi-0020090-b024] Yon JM, Perahia D, Ghelis C (1998). Conformational dynamics and enzyme activity. Biochimie.

[pcbi-0020090-b025] Berendsen HJC, Hayward S (2000). Collective protein dynamics in relation to function. Curr Opin Struct Biol.

[pcbi-0020090-b026] Hinsen K (1998). Analysis of domain motions by approximate normal mode calculations. Proteins.

[pcbi-0020090-b027] Hinsen K, Thomas A, Field MJ (1999). Analysis of domain motions in large proteins. Proteins.

[pcbi-0020090-b028] Wriggers W, Mehler E, Pitici F, Weinstein H, Schulten K (1998). Structure and dynamics of calmodulin in solution. Biophys J.

[pcbi-0020090-b029] Wilson MA, Brunger AT (2003). Domain flexibility in the 1.75 A resolution structure of Pb2+-calmodulin. Acta Crystallogr D Biol Crystallogr.

[pcbi-0020090-b030] Wilson MA, Brunger AT (2000). The 1.0 A crystal structure of Ca(2+)-bound calmodulin: An analysis of disorder and implications for functionally relevant plasticity. J Mol Biol.

[pcbi-0020090-b031] Ikura M, Clore GM, Gronenborn AM, Zhu G, Klee CB (1992). Solution structure of a calmodulin-target peptide complex by multidimensional NMR. Science.

[pcbi-0020090-b032] Meador WE, Means AR, Quiocho FA (1992). Target enzyme recognition by calmodulin: 2.4 A structure of a calmodulin-peptide complex. Science.

[pcbi-0020090-b033] Beeser SA, Goldenberg DP, Oas TG (1997). Enhanced protein flexibility caused by a destabilizing amino acid replacement in BPTI. J Mol Biol.

[pcbi-0020090-b034] Battiste JL, Li R, Woodward C (2002). A highly destabilizing mutation, G37A, of the bovine pancreatic trypsin inhibitor retains the average native conformation but greatly increases local flexibility. Biochemistry.

[pcbi-0020090-b035] Zaman MH, Shen MY, Berry RS, Freed KF, Sosnick TR (2003). Investigations into sequence and conformational dependence of backbone entropy, inter-basin dynamics and the Flory isolated-pair hypothesis for peptides. J Mol Biol.

[pcbi-0020090-b036] Eddy SR (1995). Multiple alignment using hidden Markov models. Proc Int Conf Intell Syst Mol Biol.

[pcbi-0020090-b037] Karplus K, Barrett C, Hughey R (1998). Hidden Markov models for detecting remote protein homologies. Bioinformatics.

[pcbi-0020090-b038] Burgering MJM, Hald M, Boelens R, Breg JN, Kaptein R (1995). Hydrogen-exchange studies of the arc repressor: Evidence for a monomeric folding intermediate. Biopolymers.

[pcbi-0020090-b039] Peng XD, Jonas J, Silva JL (1993). Molten-globule conformation of arc repressor monomers determined by high-pressure H-1-NMR spectroscopy. Proc Natl Acad Sci U S A.

[pcbi-0020090-b040] Silva JL, Silveira CF, Correia A, Pontes L (1992). Dissociation of a native dimer to a molten globule monomer: Effects of pressure and dilution on the association equilibrium of arc repressor. J Mol Biol.

[pcbi-0020090-b041] Bowie JU, Sauer RT (1989). Equilibrium dissociation and unfolding of the arc repressor dimer. Biochemistry.

[pcbi-0020090-b042] Brown BM, Bowie JU, Sauer RT (1990). Arc repressor is tetrameric when bound to operator DNA. Biochemistry.

[pcbi-0020090-b043] Bowie JU, Sauer RT (1989). Identifying determinants of folding and activity for a protein of unknown structure. Proc Natl Acad Sci U S A.

[pcbi-0020090-b044] Zagorski MG, Bowie JU, Vershon  aK, Sauer RT, Patel DJ (1989). NMR-studies of arc repressor mutants: Proton assignments, secondary structure, and long-range contacts for the thermostable proline-8-leucine variant of arc. Biochemistry.

[pcbi-0020090-b045] Knight KL, Sauer RT (1989). DNA-binding specificity of the arc and mnt repressors is determined by a short region of N-terminal residues. Proc Natl Acad Sci U S A.

[pcbi-0020090-b046] Vershon AK, Bowie JU, Karplus TM, Sauer RT (1986). Isolation and analysis of arc repressor mutants: Evidence for an unusual mechanism of DNA binding. Proteins.

[pcbi-0020090-b047] Breg JN, van Opheusden JH, Burgering MJ, Boelens R, Kaptein R (1990). Structure of Arc repressor in solution: Evidence for a family of beta-sheet DNA-binding proteins. Nature.

[pcbi-0020090-b048] Cheng XD, Balendiran K, Schildkraut I, Anderson JE (1994). Structure of PvuII endonuclease with cognate DNA. EMBO J.

[pcbi-0020090-b049] Spyridaki A, Matzen C, Lanio T, Jeltsch A, Simoncsits A (2003). Structural and biochemical characterization of a new Mg2+ binding site near Tyr94 in the restriction endonuclease PvuII. J Mol Biol.

[pcbi-0020090-b050] Horton JR, Nastri HG, Riggs PD, Cheng X (1998). Asp34 of PvuII endonuclease is directly involved in DNA minor groove recognition and indirectly involved in catalysis. J Mol Biol.

[pcbi-0020090-b051] Syed RS, Reid SW, Li CW, Cheetham JC, Aoki KH (1998). Efficiency of signalling through cytokine receptors depends critically on receptor orientation. Nature.

[pcbi-0020090-b052] Dube S, Fisher JW, Powell JS (1988). Glycosylation at specific sites of erythropoietin is essential for biosynthesis, secretion, and biological function. J Biol Chem.

[pcbi-0020090-b053] Narhi LO, Arakawa T, Aoki KH, Elmore R, Rohde MF (1991). The effect of carbohydrate on the structure and stability of erythropoietin. J Biol Chem.

[pcbi-0020090-b054] Dordal MS, Wang FF, Goldwasser E (1985). The role of carbohydrate in erythropoietin action. Endocrinology.

[pcbi-0020090-b055] Darling RJ, Kuchibhotla U, Glaesner W, Micanovic R, Witcher DR (2002). Glycosylation of erythropoietin affects receptor binding kinetics: Role of electrostatic interactions. Biochemistry.

[pcbi-0020090-b056] Wen D, Boissel JP, Showers M, Ruch BC, Bunn HF (1994). Erythropoietin structure-function relationships. Identification of functionally important domains. J Biol Chem.

[pcbi-0020090-b057] Narhi LO, Aoki KH, Philo JS, Arakawa T (1997). Changes in conformation and stability upon formation of complexes of erythropoietin (EPO) and soluble EPO receptor. J Prot Chem.

[pcbi-0020090-b058] Cheetham JC, Smith DM, Aoki KH, Stevenson JL, Hoeffel TJ (1998). NMR structure of human erythropoietin and a comparison with its receptor bound conformation. Nat Struct Biol.

[pcbi-0020090-b059] Elliott S, Lorenzini T, Chang D, Barzilay J, Delorme E (1997). Mapping of the active site of recombinant human erythropoietin. Blood.

[pcbi-0020090-b060] Romero P, Obradovic Z, Kissinger C, Villafranca JE, Dunker AK (1997). Identifying disordered regions in proteins from amino acid sequences. Proc IEEE Int Conf Neural Networks.

[pcbi-0020090-b061] Jones DT, Ward JJ (2003). Prediction of disordered regions in proteins from position specific score matrices. Proteins.

[pcbi-0020090-b062] Linding R, Jensen LJ, Diella F, Bork P, Gibson TJ (2003). Protein disorder prediction: Implications for structural proteomics. Structure.

[pcbi-0020090-b063] Yang ZR, Thomson R, McNeil P, Esnouf RM (2005). RONN: The bio-basis function neural network technique applied to the detection of natively disordered regions in proteins. Bioinformatics.

[pcbi-0020090-b064] Linding R, Russell RB, Neduva V, Gibson TJ (2003). GlobPlot: Exploring protein sequences for globularity and disorder. Nucleic Acids Res.

[pcbi-0020090-b065] Prilusky J, Felder CE, Zeev-Ben-Mordehai T, Rydberg E, Man O (2005). FoldIndex(C): A simple tool to predict whether a given protein sequence is intrinsically unfolded. Bioinformatics.

[pcbi-0020090-b066] Dosztanyi Z, Csizmok V, Tompa P, Simon I (2005). The pairwise energy content estimated from amino acid composition discriminates between folded and intrinsically unstructured proteins. J Mol Biol.

[pcbi-0020090-b067] Liu J, Rost B (2003). NORSp: Predictions of long regions without regular secondary structure. Nucleic Acids Res.

[pcbi-0020090-b068] Oldfield CJ, Cheng Y, Cortese MS, Romero P, Uversky VN (2005). Coupled folding and binding with alpha-helix-forming molecular recognition elements. Biochemistry.

[pcbi-0020090-b069] Schlessinger A, Rost B (2005). Protein flexibility and rigidity predicted from sequence. Proteins.

[pcbi-0020090-b070] Berman HM, Westbrook J, Feng Z, Gilliland G, Bhat TN (2000). The Protein Data Bank. Nucleic Acids Res.

[pcbi-0020090-b071] Wang G, Dunbrack RL (2003). PISCES: A protein sequence culling server. Bioinformatics.

[pcbi-0020090-b072] Altschul S, Madden T, Schaffer A, Zhang JH, Zhang Z (1998). Gapped BLAST and PSI-BLAST: A new generation of protein database search programs. FASEB J.

[pcbi-0020090-b073] Li WZ, Jaroszewski L, Godzik A (2002). Sequence clustering strategies improve remote homology recognitions while reducing search times. Prot Eng.

[pcbi-0020090-b074] Li WZ, Jaroszewski L, Godzik A (2001). Clustering of highly homologous sequences to reduce the size of large protein databases. Bioinformatics.

[pcbi-0020090-b075] Li WZ, Jaroszewski L, Godzik A (2002). Tolerating some redundancy significantly speeds up clustering of large protein databases. Bioinformatics.

[pcbi-0020090-b076] Henrick K, Thornton JM (1998). PQS: A protein quaternary structure file server. Trends Biochem Sci.

[pcbi-0020090-b077] Haliloglu T, Bahar I, Erman B (1997). Gaussian dynamics of folded proteins. Phys Rev Lett.

[pcbi-0020090-b078] Flory PJ (1976). Statistical thermodynamics of random networks. Proc Math Phys Eng Sci.

[pcbi-0020090-b079] Tirion MM (1996). Large amplitude elastic motions in proteins from a single-parameter, atomic analysis. Phys Rev Lett.

[pcbi-0020090-b080] Iglewicz B, Hoaglin DC (1993). How to detect and handle outliers.

[pcbi-0020090-b081] Joachims T (1999). Making large-scale SVM learning practical. In Scholkopf B, Burges C, Smola A (eds). Advances in kernel methods: Support vector learning.

[pcbi-0020090-b082] Palliser CC, Parry DA (2001). Quantitative comparison of the ability of hydropathy scales to recognize surface beta-strands in proteins. Proteins.

